# Familial occurrence of imperforate hymen in premature monozygotic twins and their mother: a case report and literature review

**DOI:** 10.3389/fped.2024.1377290

**Published:** 2024-07-19

**Authors:** Bernadine Han Ern Chua, Zubair Amin, Yvonne Peng Mei Ng

**Affiliations:** ^1^ MOH Holdings, Ministry of Health, Singapore, Singapore; ^2^ Department of Neonatology, Khoo Teck Puat-National University Children’s Medical Institute, National University Health System, Singapore, Singapore; ^3^ Department of Paediatrics, Yong Loo Lin School of Medicine, National University of Singapore, Singapore, Singapore

**Keywords:** autosomal dominant, X-linked dominant, inheritance, neonate, congenital malformation

## Abstract

**Background:**

Imperforate hymen is an uncommon obstructive anomaly of the developing female reproductive tract. There are occasional case reports of imperforate hymen occurring in family clusters, suggesting a plausible familial mode of inheritance. We describe a set of monozygotic premature twins with imperforate hymen noted at birth, whose mother was diagnosed with the same condition as a teenager. We also elucidate the likely underlying mode of inheritance of imperforate hymen.

**Method:**

We utilized the CARE (Case Report) guideline in reporting the cases.

**Case presentation:**

These are monozygotic twins born prematurely at 30 weeks of gestation, noted at birth to have bulging cyst-like structures protruding from their vaginas. The twins were not dysmorphic and did not have any other congenital malformations. Over the next few weeks, these cyst-like structures (mucoceles) became less prominent. The genital anomaly was diagnosed as imperforate hymen. Their mother was also diagnosed with an imperforate hymen when she was 12 years old and was treated with hymenectomy.

**Discussion:**

This unique occurrence of imperforate hymen in a set of premature monozygotic twins and their mother suggests a plausible autosomal or X-linked dominant mode of inheritance. Given the role of genetic inheritance in imperforate hymen development, it is important to screen female relatives of an index case for this genital anomaly.

## Introduction

Imperforate hymen results from the failed degeneration of the hymenal epithelial cells at 22 weeks of gestation (1–3). Embryologically, the hymen is the junction of the urogenital sinus and sinovaginal bulbs and derives from invaginations of the posterior wall of the urogenital sinus (4). It is postulated that an imperforate hymen occurs when the hymen fails to canalize with the rest of the vagina when the sinovaginal bulbs canalize at the site where the uterovaginal canal meets the urogenital sinus (2, 3).

The reported incidence of imperforate hymen is approximately 0.05%–0.1% of females, making it an uncommon obstructive anomaly of the developing female reproductive tract (5–7). Its occurrence is usually sporadic, with rare reports of non-syndromic familial cases (5). The authors of several reports of familial clusters have suggested autosomal-recessive, autosomal-dominant, and X-linked dominant modes of inheritance (5–8).

We describe a set of premature monozygotic twins with imperforate hymen noted at birth and their mother, who was treated for the same condition as a teenager. We also elucidate the possible underlying mode of inheritance of imperforate hymen and provide an update on the literature.

## Methods

We followed the CARE (Case Report) Guideline in reporting this case (9) (Supplementary Material S1). We obtained parental written informed consent to publish this case report and have included the family's perspectives in this manuscript. There is no identifying information included in this report.

## Case reports

The twins were naturally conceived. The parents are of Malay ethnicity and are non-consanguineous. This was the first pregnancy of the mother. Antenatal scans revealed that the fetuses were monozygotic diamniotic twins with no anomalies detected. The amniotic membranes of the mother spontaneously ruptured at 30 weeks and 6 days of pregnancy. Her labor progressed rapidly, and she delivered the twins vaginally.

**Figure 1:**
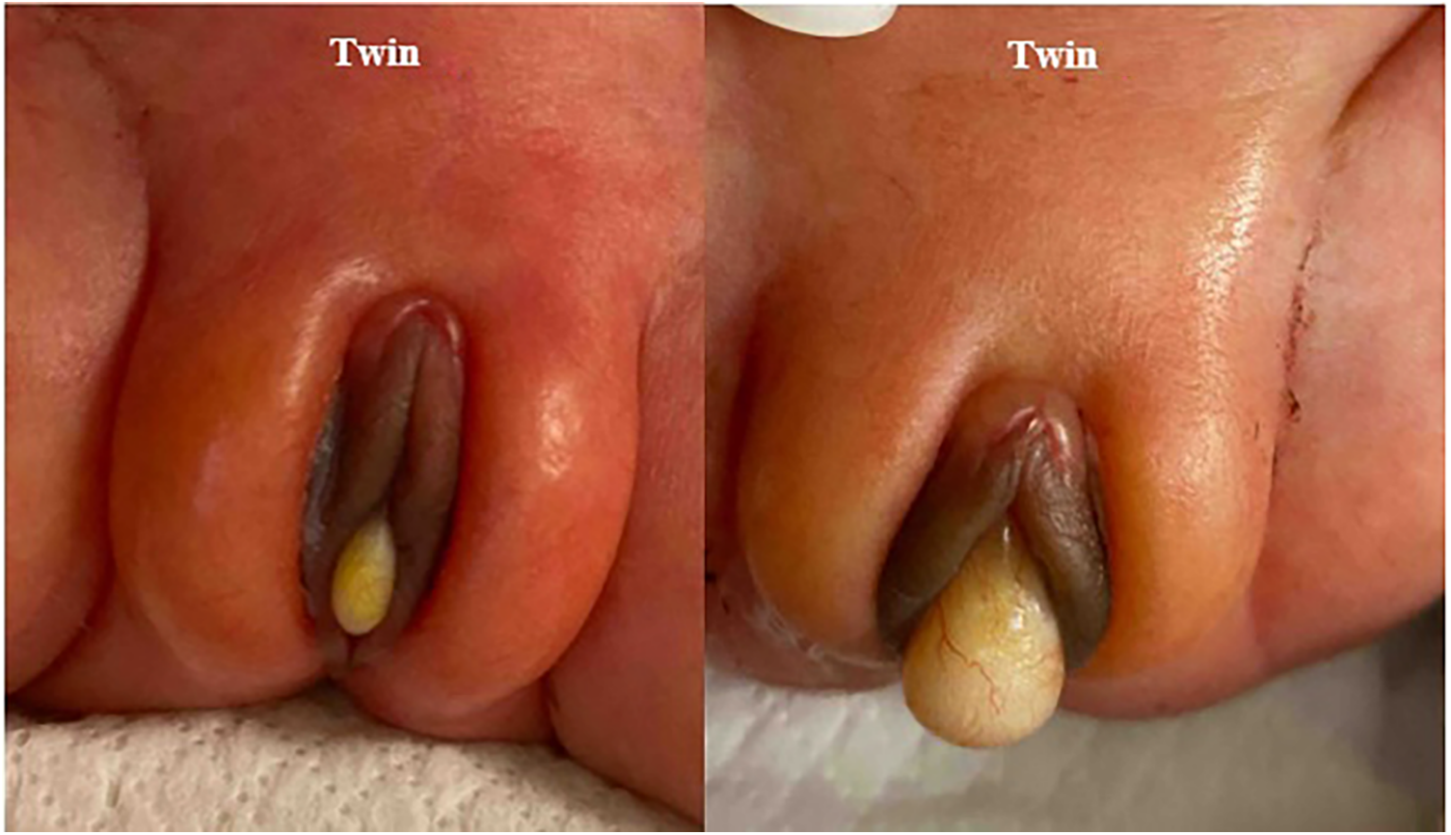
Bulging mucoceles in both twins at birth.

**Figure 2:**
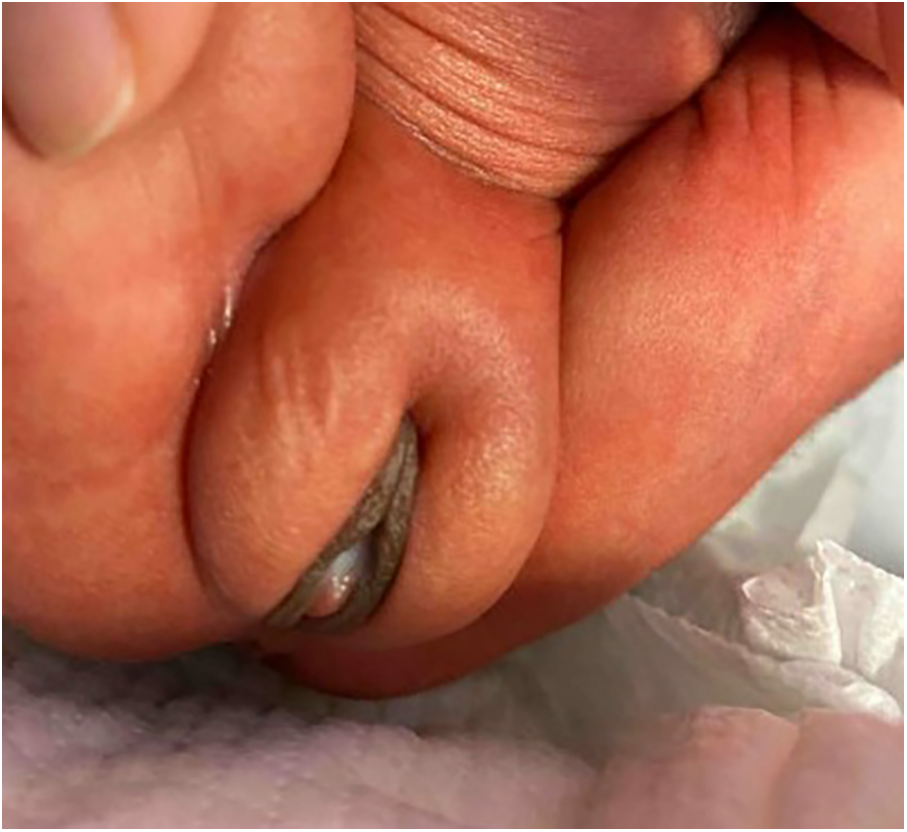
Appearance of imperforate hymen around day 10.

At birth, both twins were noted to have a bulging cyst-like structure protruding from their vaginas, which was later recognized as a mucocele (Figure 1). No other dysmorphisms or congenital malformations were noted in the twins. Over the next few weeks, the mucocele in each twin became less prominent in appearance (Figure 2). No specific diagnostic test was performed. This was diagnosed as imperforate hymen and managed expectantly without any intervention.

At their recent follow-up at 2.5 months of age, both twins were healthy, and their hymens remained imperforate. The parents have been counseled to anticipate the possible complications of imperforate hymen around puberty, which are lower abdominal pain, urinary retention, and primary amenorrhea.

The twins’ mother was the first member in her family diagnosed with an imperforate hymen at 12 years of age. No female relatives of the twins’ father have been diagnosed with imperforate hymen.

## Discussion

We conducted a literature search on Medline in December 2023 using MeSH keywords “Hymen” and “Inheritance Pattern” and found no previously reported similar cases. Thus, this is the first report of a set of monozygotic twins and their mother with imperforate hymen. This report is also unique as imperforate hymen has rarely been reported in premature infants.

The first familial case of imperforate hymen was reported by McIlroy and Ward in 1930, involving three otherwise healthy sisters who underwent surgical correction for the condition (10). Subsequently, other authors have reported cases of imperforate hymen in family members. Watrowski et al., in a review in 2013, found eight cases of familial occurrence of imperforate hymen in the literature (11). More recently, Baanitse et al. reported three sisters who presented with abdominal pain at different ages (8, 6, and 33 months old) and were diagnosed with imperforate hymen (5). Interestingly, their mother was treated for the same condition only when she reached puberty (5).

Our reported cases include a set of monozygotic (identical) twins with imperforate hymen whose mother had the same condition. This suggests a dominant mode of inheritance, which can either be autosomal or X-linked. Similarly, Stelling et al. reported a 12-year-old girl who presented with peritonitis and was screened for imperforate hymen because her mother and her mother's monozygotic twin were diagnosed with imperforate hymen at 14 years of age (1). Conversely, other studies have suggested a recessive mode of inheritance for imperforate hymen. For example, Sakalkale and Samarakkody reported two cases of imperforate hymen—a 13-year-old girl with lower abdominal pain and urinary retention and her 14-year-old maternal first cousin with cyclical lower abdominal pain (8). Watrowski et al. reported the occurrence of imperforate hymen in a set of dizygotic twins (11). Given the various possible modes of inheritance, it is likely that imperforate hymen can be caused by mutation in several genes (6).

Diagnosis of an imperforate hymen usually occurs during puberty (4), as females with this condition are asymptomatic before menarche. At puberty, they present with amenorrhea, abdominal pain, and possible urinary retention (12, 13). During the neonatal period, endogenous maternal estrogen stimulation can result in a mucocele and present as a bulging hymen, which was seen in our twins (2, 6). The mucocele usually resolves spontaneously without needing intervention, which occurred in these twins (14). Imperforate hymen has also been detected in a fetus during antenatal ultrasound studies (15).

Imperforate hymen has rarely been reported to occur in premature infants. We were able to identify another case report of imperforate hymen in a 35-week premature infant who also had duodenal atresia (16). The baby had a large swelling in the introitus with normal urethral and anal openings. The imperforate hymen was managed by hymenal incision, while the duodenal atresia was surgically corrected with duodenoduodenostomy (16).

The detection and diagnosis of an imperforate hymen can be confirmed by a simple physical examination of the genitalia at any age, without costly radiographic investigations (5, 13). Despite this, diagnosis of individuals with imperforate hymen may be delayed till puberty. This may result in abdominal pain, unnecessary radiological investigations, or endocrine evaluation for amenorrhea (4, 13).

Imperforate hymen may also occur in association with obstructed hemivagina and ipsilateral renal anomaly (OHVIRA), which is a rare Müllerian duct anomaly with uterus didelphys, unilateral obstructed hemivagina, and ipsilateral renal agenesis (17). Patients with this anomaly usually present after menarche with pelvic pain and/or an abdominal mass due to obstruction of menstrual flow. In a recent review of a series of cases of OHVIRA patients, the authors recommend regular follow-up of pre-menarche OHVIRA patients without any symptoms (17). Surgery is preferred for symptomatic patients and post-menarche patients. The authors also suggested long-term follow-up of these patients for possible renal and gynecological issues (17).

We would like to highlight some limitations of this case report. The generalizability of the findings and conclusions from this report are limited because these are based only on one set of twins and their mother. We also did not perform a genetic analysis that could identify the underlying gene mutation.

Our report gives stronger credence to earlier reports of an underlying genetic basis for the mechanisms of imperforate hymen. As imperforate hymen can happen in family clusters, we suggest proactive screening of related female family members for this uncommon but easily treatable condition.

## Data Availability

The original contributions presented in the study are included in the article/Supplementary Material, further inquiries can be directed to the corresponding author.
